# Organic Disease of the Heart*Lecture delivered at Bellevue Hospital, New York, May 18, 1883.

**Published:** 1883-09

**Authors:** Austin Flint


### Organic Disease of the Heart.*

Gentlemen—To-day I propose to give you a kind of practical exercise in diseases of the heart, and, bringing before you a number of cases, none of which I have myself examined, endeavor to call to your attention such points in diagnosis and treatment as may arise in connection with them, and which may be of service to you in your study of this important subject. The first patient being now before us, I will proceed at once to make an examination in reference to his heart, without obtaining any history or asking any questions in reference to his present condition. On looking at the man we notice, in the first place, that he has no embarrassment in breathing, and that there is no cyanosis present. In regard to his heart we note that two movements are visible upon the chest-walls, one systolic and the other diastolic. Now let us see whether the heart is enlarged or not. The apex-beat is in the sixth intercostal space, on a

* Lecture delivered at Bellevue Hospital, New York, May 18, 1883.

vertical line with the nipple, which indicates that while the organ is enlarged, the degree of enlargement is not very great. On auscultation it is found that the first sound of the heart reaches its maximum intensity at this point. The next point is to find how large a portion of the heart is uncovered with lung tissue. The normal area of cardiac dullness, as you are aware, is of the general shape of a right-angled triangle, and here we find that this area of superficial dullness is at least double the normal size. We have thus made out the undoubted existence of a certain amount of enlargement of the heart. Whether this enlargement is due to hypertrophy or to dilatation is a very simple matter to determine. If there is hypertrophy there will be increased impulse, and if dilatation, weakened impulse. It is well to remember, however, that the apex-beat may be feeble while the impulse above that is quite strong. When this is the case it is due to a change in the shape of the heart, the organ having become more globular in outline. The next point in the differentiation between the two, is to find out whether the impulse is of a heaving or sharp character. It is found to be of the latter, which results from dilatation; and we have in this case, therefore, enlargement of the heart with predominant dilatation.

In some cases enlargement of the heart is due to disease of the kidney, in which case the left side of the organ is principally affected, and in others to pulmonary emphysema, where the right, side is enlarged. As a general rule, however, this pathological condition is dependent on valvular disease of the heart. There is certainly no emphysema present in this case, and presuming that there is no kidney trouble either, we will look for evidence of disease of the valves; and as the left side of the heart is far more commonly the seat of such affections than the right, we will make our examination of that first. We may have, as you know, either regurgitation or obstruction present, or both combined, and by means of auscultation we can usually determine what the condition is without difficulty. On applying the stethoscope to the left side of the heart here I get a soft, bellows-like murmur, synchronous with the first sound of the heart, which reaches its maximum intensity at the apex, and which is also transmitted beyond the apex to the left. There is no presystolic murmur noticeable, and we decide, therefore, that there is present in this case a regurgitation from the left ventricle into the left auricle, or mitral regurgitation, as it is called. Auscultation of the aortic valves, by placing the stethoscope in the second intercostal space, just to the right of the sternum, shows a negative result. The case, therefore, is a perfectly simple one, the enlargement of the heart depending on mitral insufficiency, which always leads to more or less increase in the size of the right ventricle, as is the case here.

One point of considerable practical interest in such a case as this, is to compare the aortic second sound with the pulmonic second sound in order to form some idea of the degree of insufficiency existing. It is known that where there is hypertrophy of the right ventricle the pulmonic second sound is intensified, and in proportion, as a rule, as the second aortic sound is weakened, is the amount of mitral insufficiency present. In the present instance, I may say, there is a condition of affairs which can be tolerated almost indefinitely by the system, and I would warn you, in cardiac affections generally, not to form too hasty a prognosis of impending serious trouble.

Now, in order to make the case more complete, let us ask a few questions of the patient; but before doing so I will venture to predict, with a very considerable degree of confidence, that this man has had acute articular rheumatism at some period of his life. Yes, he says that he has had the disease, the first attack fifteen years ago, and the last, three years ago. He does not give any history of endocarditis in connection with any of his attacks; but this is a complication which frequently escapes notice altogether. Yet its effects are apt to be of a permanent and serious character, and in this case it has undoubtedly led to the organic disease which is now present. In such a condition of the heart the patient after a time finds that he suffers more or less from shortness of breath in taking exercise which formerly produced no such effect, and that is the chief trouble here. The man says he "gets out of wind," and with this exception he is really quite well. This, then, is a perfectly typical case of simple mitral insufficiency, without mitral obstruction, and without any complicating disease of the aortic valves; typical as regards, first, the physical signs present; second, the connection of the trouble with acute articular rheumatism; and third, the general symptoms.

Finally, let us inquire what are the therapeutical indications in such a case as this? Too many practitioners under these circumstances resort at once to digitalis. Given a case of disease of the heart, and digitalis is prescribed as a matter of course. But in the present instance digitalis is not called for at all. All remedies which cause depression and disturbance of the system are contra-indicated, first, because they would accomplish no good, and secondly, so far as they had any effect it would be an injurious one. What we have to do here is to see what the man needs to put him in the best general condition possible. It is of the most importance to him to have good, nourishing alimentation, and if his digestion is defective, he should have appropriate remedies to improve it. In this case there is merely a question of toleration, and as long as the general system is in good condition, the heart is not likely to give rise to trouble. The patient has now been in the hospital a week, and with the nourishing diet, rest and freedom from anxiety which he has had here, he has in this short time already improved to a very appreciable extent, as I am given to understand.

As to the matter of dyspnœa it is too much the custom for physicians to tell their patients suffering from cardiac disease that they must be exceedingly careful about taking exercise for fear of evil results; but this kind of advice has a bad effect on such patients. In the first place, it keeps their minds continually on the subject of their hearts, which of itself is very injurious, and, secondly, it deprives them of that amount of exercise which is essential to the maintenance of health, and which, indeed, as a rule, they could take perfectly well. The safest plan is to direct such patients to take just as much exercise as they can, without inconvenience or discomfort.

In the case of our second patient, whom I now bring before you, we notice on inspection of the chest only one movement instead of two, as in the other, at the apex of the heart, while there is in addition a second movement at the base. The heart is undoubtedly enlarged, because the apex-beat, as you will observe, is considerably lower down than it ought to be. Unlike the former case, it is also found much further to the left than its normal position, a point which leads us to infer the presence of mitral disease. In this respect the first case was an exception to the general rule, the apex-beat being carried so little to the left that it would, perhaps, seem to indicate an aortic rather than a mitral affection. On account of the strength of the impulse in the present instance, we infer that there is hypertrophy rather than dilatation of the heart.

On auscultation we get a systolic murmur at the apex which differs from that in the other case in being less loud, much lower in pitch, and not of such a marked bellows-like character. Again, however, there is no evidence of any mitral obstruction, but when we apply the stethoscope at the base we get with each systole a loud, rough murmur, which must therefore be a direct aortic murmur. But in addition, instead of the second sound of the heart, there is found another rough murmur, so that we have here a systolic and diastolic aortic murmur besides the systolic mitral murmur. The latter is much weaker in tone than the aortic murmurs, but notwithstanding this, it is quite possible that the mitral disease may be more serious than the aortic. The practical lesson to be learned from this is that it is never safe to judge of the gravity of disease of the heart from the intensity and quality of the murmurs heard. I find in this case distinct evidencc of dilatation of the aorta (though not necessarily in the form of a sacculated aneurism) in addition to the cardiac disease. I would warn you, however, never to form a diagnosis of aortic aneurism from the roughness of murmurs heard at the base.

This patient is only twenty-eight, and therefore not of an age which entitles him to have aneurism, which, as a rule, occurs at a later period of life. I am quite sure, however, that there is dilatation of the aorta here, and when this occurs as early as the age of this man it is almost invariably due to one cause, namely, syphilis. This he denies having had, but as he also says that he has never had acute articular rheumatism I am afraid we shall have to place the weight of pathological authority against the word of the patient.

On questioning him, I find that notwithstanding the fact that he has an enlarged heart, with both mitral and aortic lesions, he is able to work hard, and that he manages to get along very well altogether with the exception of a pain in his side sometimes. His heart is really much larger than that of the other patient, but he suffers less inconvenience because the valvular disease is compensated for by predominant hypertrophy, while in the other case the heart has reached the limit of its growth. In the latter the cardiac walls have become more or less thinned, and the predominant dilatation gives rise to greater weakness and interference with respiration.

Our next patient is a Bohemian, who does not speak any English. On inspection and palpation we get two impulses, and find that the one at the apex is not so strong as a second one that is higher up. The apex being lowered and carried considerably to the left in this case also, we have a sufficient basis to warrant the guess that here again there is a mitral lesion. Auscultation reveals the presence of a loud, high-pitched, bellows-like systolic murmur, and there being no presystolic murmur, we form the diagnosis of mitral regurgitation without obstruction, as in the other cases. At the base, the only adventitius sound is an indistinct murmur, which is simply a transmission of that heard at its maximum intensity at the apex. We have again, therefore, a case of mitral insufficiency and enlargement of the heart, and as it is the right ventricle that is principally affected in the latter, the pulmonic second sound ought to be intensified as compared with the aortic second sound. I find, in fact, that the difference between the two is so great that it convinces me that it depends on weakness of the aortic sound as well as the intensifying of the pulmonic, and therefore we are enabled to form some idea of the amount of regurgitation that is here present.

In the last patient whom we have time to examine to-day, I find again the apex lowered and carried considerably to the left. There is, then, cardiac enlargement here also, and from the character of the impulse there can be but little doubt that there is predominant hypertrophy rather than dilatation. The first sound of the heart also indicates this. In hypertrophy the first sound is loud, long, and booming, while in dilatation it is short, weak, and valvular in character. There is once more a mitral systolic or regurgitant murmur, and no direct mitral murmur indicating obstruction. In this case it is quite a feeble murmur, and therefore in marked contrast to that heard in some of the other patients. At the base there is found an aortic direct murmur, loud and rough, and an aortic regurgitant, feeble and soft. In connection with this case, I would again impress upon you the caution of not judging of the gravity of cardiac disease from the intensity of the murmurs present. Twenty years ago, there was connected with the house-staff of this hospital, a young physician who had a well-marked aortic regurgitant murmur, yet ever since then he has continued in the active practice of his profession, and to-day the general state of his health is excellent. If I had time, I could tell you of a large number of similar instances. Although this patient has enlargement of the heart, mitral insufficiency, and both aortic insufficiency and obstruction, I will venture to predict that he will do well, because his system has already tolerated this cardiac condition for a considerable time, and the symptoms of the case do not now indicate any immediate serious trouble such as we would apprehend if there were present dyspnœa without taking exercise, cyanosis, and general dropsy.—*Prof. Austin Flint, M. D., in Boston Medical and Surgical Journal.*

